# Nanometer-Scale Cavities for Mid-Infrared Radiation via Image Phonon Polariton Resonators

**DOI:** 10.1021/acs.nanolett.5c01352

**Published:** 2025-05-19

**Authors:** Michael Klein, Yonatan Gershuni, Alisa Perutski, Jean-Paul Hugonin, Itai Epstein

**Affiliations:** † School of Electrical Engineering, Faculty of Engineering, 26745 Tel Aviv University, Tel Aviv 6997801, Israel; ‡ Laboratoire Charles Fabry, Institut d’Optique Graduate School, 226352 Universite Paris-Saclay, CNRS, 91127 Palaiseau, France; ¶ Center for Light−Matter Interaction, Tel Aviv University, Tel Aviv 6997801, Israel; § QuanTAU, Quantum Science and Technology Center, Tel Aviv University, Tel Aviv 6997801, Israel

**Keywords:** phonon polaritons, polar dielectrics, image polaritons, mid-infrared, resonators

## Abstract

Surface polaritons play a pivotal role in strong light–matter interactions at the nanoscale due to their ability to confine light to deep-subwavelength dimensions. A promising class of materials exhibiting such a polaritonic response is polar dielectrics, which support surface phonon polaritons (SPhPs). While SPhPs offer significantly lower losses compared to other polaritons, their potential has been underutilized due to their limited ability to reach large confinement factors. Here, we demonstrate a system composed of silver nanocubes deposited on a SiC polar dielectric, which experimentally realizes the antisymmetric-image-phonon-polariton mode, a hybridized SPhP mode that can confine mid-infrared radiation to extremely small mode volumes, almost a billion times smaller than their free-space volume, with quality factors an order-of-magnitude greater than those of surface plasmons or graphene plasmons, surpassing values of 180. Our method is general, scalable, and applicable to any polar dielectric, opening the path for controlling and manipulating strong light–matter interactions at the nanoscale in the long-wavelength range.

## Graphical abstract

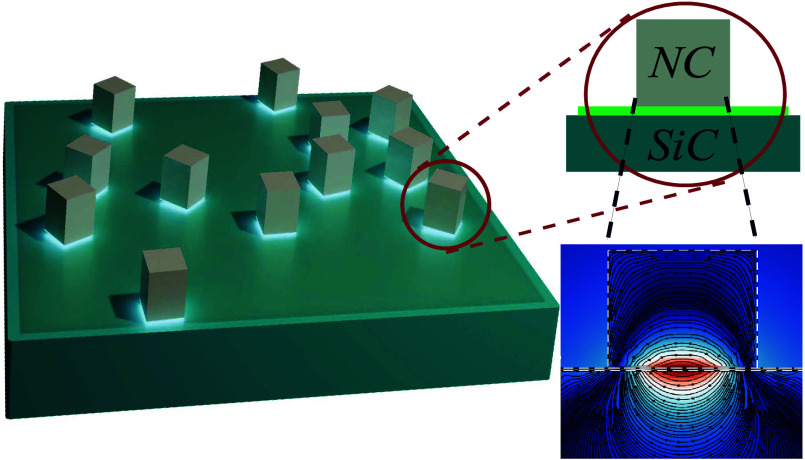

Surface polaritons (SPs) are propagating electromagnetic modes that arise from the coupling between electromagnetic radiation and a material excitation in the form of an electric dipole.^1–4^ Fundamentally, they can be described as propagating waves that are confined to the interface between two materials, where one possesses a positive real part of the permittivity and the other a negative real part, such as surface plasmon polaritons (SPPs) and graphene plasmons (GPs).^1,5^ When the supporting material is hyperbolic, it can give rise to hyperbolic phonon polaritons (HPhPs) with similar properties.^6–11^ SPs play an important role in strong light–matter interactions at the nanoscale due to their ability to confine light to deep-subwavelength dimensions, which is accompanied by enhancement of the optical field.^1,12–15^ By exploitation of these attributes, a wide range of applications have been demonstrated, such as molecular spectroscopy, narrow-band thermal emitters, thin-film sensing, biosensing, and more.^16–19^

The momentum of SPs can be further increased by placing the polaritonic material near a metallic surface.^20–31^ The latter imitates the presence of the polaritonic surface through image charges and is thus commonly termed “image polariton”.^28,30,32,33^ This configuration supports two modes in terms of the electric field distribution: symmetric and antisymmetric. The symmetric one carries smaller momentum compared to the SP, while the antisymmetric image polariton (AIP) has been shown to carry extremely large momentum, with wavelength confinement that is several orders of magnitude smaller than the free-space wavelength.^22,27,28,31,34–39^

The large momentum carried by SPs, HPhPs, and AIPs also introduces a significant momentum mismatch between the polariton and the free-space photon,^1,40–42^ thus making their excitation and observation challenging. Several approaches have been previously utilized to overcome this issue, such as using patterned structures^28,31,43,44^ or scattering scanning-near-field-optical microscopy (s-SNOM),^25,45–49^ and have also been successfully applied to measure surface-plasmon AIPs, graphene-plasmon AIPs, and hyperbolic-phonon-polariton AIPs.^20,24–26,28–30,50,51^

Another promising system that exhibits negative permittivity and thus can support SPs is polar dielectrics. In these materials, the coupling of charged lattice ions with electromagnetic radiation gives rise to surface phonon polaritons (SPhPs).^2,44,45,49,52–54^ Since their pioneering realization and measurements by s-SNOM,^43,45,52,55^ SPhPs have been utilized for thin-film biological sensing, and super-resolution imaging, for example.^17,56,57^ However, while SPhPs provide inherently low losses,^2,55^ they have not been fully exploited due to their limited ability to reach large confinement factors compared to other SPs.

In this work, we demonstrate extremely confined SPhPs via the experimental realization of their antisymmetric image polariton mode, i.e., the antisymmetric image phonon polariton (AIPhP). We achieve this by realizing AIPhP resonators in a system composed of nanometer-sized silver cubes deposited on a SiC polar dielectric. We find that the AIPhP resonators can confine mid-infrared (MIR) radiation to extremely small mode volumes, which are almost a billion times smaller than their free-space mode volume. Moreover, we show that due to their inherently low losses, AIPhPs exhibit quality factors that are an order of magnitude greater than those of surface-plasmon AIPs and graphene-plasmon AIPs, surpassing values of 180. Finally, we demonstrate that the spectral response and intensity of the AIPhP resonators can be precisely controlled by adjusting the size and concentration of the nanocubes.

Polar dielectrics, such as SiC, for example, exhibit a negative-real-part permittivity in the spectral range between the transverse optical (TO) and longitudinal optical (LO) phonon frequencies, known as the Reststrahlen band.^2,55^ A signature of this property is the large reflectivity observed in the Reststrahlen band’s spectral range, stemming from the material’s metal-like behavior. Although SPhPs carry smaller momentum compared to GPs and HPhPs, they can provide reduced propagation losses; thus, it is worthwhile to realize their AIP equivalent.

To evaluate the optical performance of SPhPs in comparison to AIPhPs, it is convenient to define the confinement factor, *k*/*k*_0_, and loss figure of merit, *Q*_p_ = Re­(*k*_p_)/Im­(*k*_p_).^48,58^ Figure 1 shows the simulated values (see the Supporting Information, SI) for SPhPs supported at the interface between semi-infinite SiC and a dielectric (*n* = 1.4; Figure 1a,b) and those for AIPhPs supported in a SiC/dielectric/Ag structure with a dielectric spacer of thickness *d* (Figure 1c,d). It can be seen in Figure 1a,b that SPhPs indeed exhibit low confinement factors nearing the lightline, *k*_0_ = ω/*c* (black curve), accompanied by large values of *Q*_p_ due to their low losses. In contrast, parts c and d of Figure 1 show that AIPhPs realized by bringing the SiC to a distance *d* from an Ag surface yield extremely large confinement factors, still with relatively low losses. As *d* decreases, the momentum increases by up to 2 orders of magnitude, and the dispersion becomes approximately linear, a characteristic behavior of AIP modes.^22,27,31,36^ Despite the increased losses at smaller *d* separations, at optimal conditions (875 cm^–1^), the confinement factor exceeds 350, with *Q*_p_ values reaching 27 for the smallest spacer thickness.

**Figure 1:**
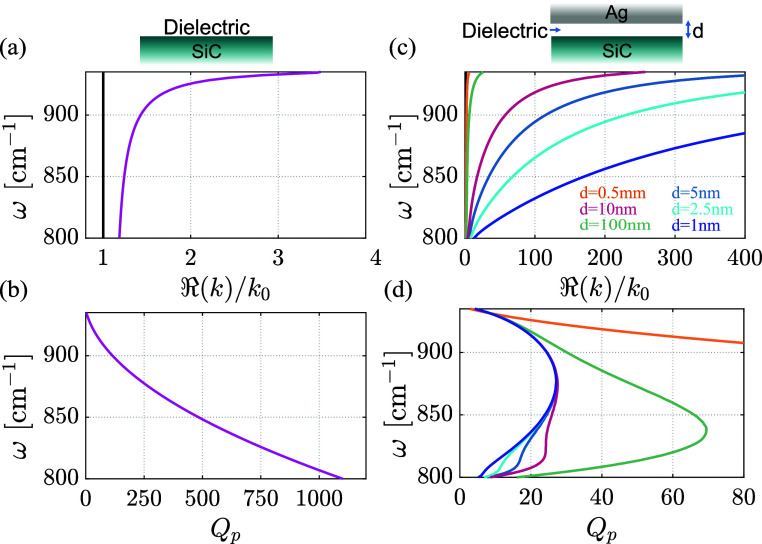
Confinement factor *k*/*k*_0_ and loss figure of merit *Q*_p_ of SPhPs and AIPhPs. (a) Calculated *k*/*k*_0_ compared to the lightline (black curve) and (b) *Q*_p_ of SPhPs at the interface between semi-infinite SiC and a dielectric within the Reststrahlen band spectral range. (c) Calculated *k*/*k*_0_ and (d) *Q*_p_ of AIPhPs supported in a SiC/dielectric/Ag structure with a dielectric spacer of thickness *d*. Both geometries are shown at the top part of the figure.

To realize such extremely confined AIPhPs, we start first by characterizing the 4H-SiC crystal at hand. Figure 2a presents optical characterization of the SiC crystal in the Reststrahlen band’s spectral range via reflection (blue curve) and Raman (red curve) measurements. The Raman curve clearly shows the locations of the TO and LO phonons, and the expected large reflectivity in this range can be observed. The dashed black curve in Figure 2a shows the reflection spectrum obtained from the transfer-matrix-method simulation of the structure (see the SI), with the permittivity of the SiC shown in Figure 2b modeled by the Lorentz model for polar materials:^55^

1 $$\epsilon \left(\omega \right)=\epsilon_{\infty }\left(\frac{{\omega_{\mathrm{LO}}}^{2}-\omega^{2}-i\Gamma_{\mathrm{LO}}\omega }{{\omega_{\mathrm{TO}}}^{2}-\omega^{2}-i\Gamma_{\mathrm{TO}}\omega }\right)$$

where ϵ_∞_ is the high-frequency permittivity, ω_LO_ and ω_TO_ are the LO and TO frequencies, respectively, and Γ_LO_ and Γ_TO_ are the phonon’s damping rates.

**Figure 2:**
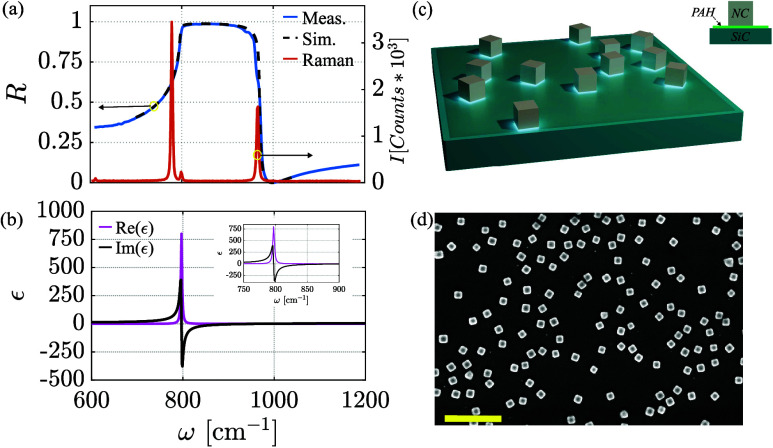
Characterization and analysis of the 4H-SiC substrate. (a) FTIR reflection spectrum (blue curve) and Raman spectrum (red curve) of the 4H-SiC material, showing the Reststrahlen band spectral range and the LO and TO phonons. The spectrum was calculated and fitted (black dashed curve) using eq 1. (b) Real (black curve) and imaginary (magenta curve) parts of the permittivity of 4H-SiC, showing the correlation between the negative real part of the permittivity to the Reststrahlen band in part a. Inset: SiC permittivity around its Reststrahlen band. (c) Sample configuration showing the 4H-SiC substrate, coated with a PAH layer, and covered with nanometer-sized silver cubes. The inset shows the cross section of a single SiC/NC structure. (d) SEM image of 100 nm silver NCs randomly dispersed on top of the 4H-SiC substrate (scale bar, 600 nm).

From the simulation fit of eq 1 to the measured reflection spectrum, we can extract the ω_LO_ = 971 cm^–1^, ω_TO_ = 796 cm^–1^, Γ_LO_ = 3 cm^–1^, and Γ_TO_ = 2.9 cm^–1^ values, with ϵ_∞_ = 6.56 ^59^ is presented in Figure 1b (dashed black curve) and shows good agreement. For completeness, the obtained complex permittivity values are presented in Figure 2b.

Because it is not practical to bring a metallic surface close to a SiC crystal, for the AIPhPs realization, we cover the bare SiC substrate with a thin layer of poly­(allylamine hydrochloride) (PAH), followed by the deposition of 100 nm silver nanocubes (NCs; see Methods). Figure 2c shows an illustration of the device where NCs are randomly scattered on top of a SiC crystal and a thin layer of PAH between them. The top face of the device can be seen in the scanning electron microscopy (SEM) image (Figure 2d), illustrating the random positions and orientations of the silver NCs.

Next, we analyze the optical response of our device within the spectral range of the Reststrahlen band. The reflection measurements with and without the NCs are shown for comparison in Figure 3a. A single dip can be seen at a frequency of 870 cm^–1^, marked by the vertical dashed gray line, when the NCs are present (blue curve), which is not observed in the measurement of the bare SiC (black line). In order to clearly separate the resonance from the large reflective background of the Reststrahlen band, we plot in Figure 3a the reflectance contrast (orange curve), defined as Δ*R*/*R*, where Δ*R* = *R* – *R*_w/NC_ and *R*_w/NC_ and *R* are the reflections measured with and without the NCs, respectively. A distinct resonant peak in the reflection contrast can be observed in Figure 3a, at the 870 cm^–1^ frequency. We note that an additional peak can also be observed at the 930 cm^–1^ frequency, indicated by the gray area in Figure 3a. This broader peak results from the rapid decrease and small changes in the reflection curves close to the edge of the Reststrahlen band and is thus not associated with the AIPhPs resonance.

**Figure 3:**
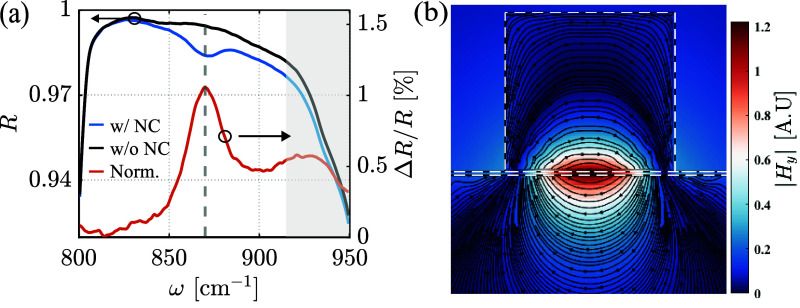
Characterization of the spectra obtained from the device comprising NCs deposited on SiC. (a) FTIR reflection measurements of the SiC substrate with (blue curve) and without (black curve) 100 nm silver NCs. The reflection contrast measurement (orange curve) illustrates the isolated resonance response of the structure. The gray dashed vertical line highlights the peak location, and the gray area covers the broader peak resulting from the Reststrahlen band’s tail. (b) 3D simulations of the magnetic field distribution over a single resonator, |*Hy*|, superimposed with the electric field lines, showing the generation of a magnetic-dipole resonance at the SiC/NC interface at resonance. In these simulations, vacuum was used as the environment and a PAH spacer thickness of 1 nm.

To understand the nature of the observed resonance, we perform three-dimensional simulations of a single SiC/NC resonator excited by far-field radiation (see Methods).^60,61^ The magnetic field distribution obtained at the resonance frequency (870 cm^–1^) over the entire structure is presented in Figure 2b, superimposed with the electric field lines. It can be seen that the electric field lines form a loop encircling an area with a strong magnetic field at the SiC/NC interface. This behavior is a signature of the AIP, as has been previously observed in surface-plasmon AIPs and graphene-plasmon AIPs.^20,27,62^ From this, we can conclude that the nature of the observed resonance is the excitation of AIPhPs, where each NC together with the SiC acts as a single AIPhP resonator. The principle of operation is that of a patch antenna, in which a rectangular metallic patch above a metallic surface creates similar magnetic-dipole resonances,^20,27^ where in our case, the negative permittivity of the SiC acts as a metal-like surface within the Reststrahlen band’s spectral range.

Next, we examine the spectral responses of the AIPhP resonators of different NC sizes and concentrations. The measured normalized reflections of three different samples with NC sizes of 100 nm (blue curve), 75 nm (orange curve), and 55 nm (yellow curve) are presented in Figure 4a. It is notable that the AIPhP resonance shifts to higher frequencies with decreasing NC size, as expected from the dispersive nature of the AIPhP resonance.^27^ To further characterize the optical response, we conducted measurements of the reflection from two additional samples with 100 nm cube size but differing NC concentrations (Figure 4b), one with approximately twice the NC density of the other (see the SI). It can be seen from the measurements that the resonance frequency remains constant in both samples because it is the NC size that sets the resonance frequency, while the amplitude of the response changes only with the concentration, with a greater concentration corresponding to a larger amplitude. These results agree well with previous observations of graphene-plasmon AIPs and surface-plasmon AIPs.^20,27^ We note that the reflection measurement captures the collective response of many resonators within the beam’s spot, resulting in the measurement of the averaged response, stemming from the nanocubes’ shape and size variations, together with their possible deformation and aggregation after deposition on the SiC surface.

**Figure 4:**
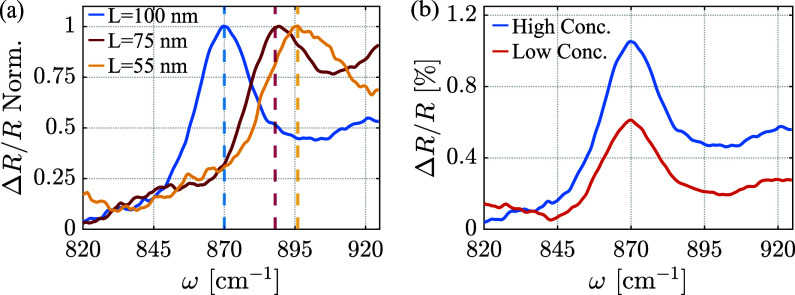
Analysis of the change in the spectral response of the AIPhP resonators with (a) a change in the NCs size (*L* = 55, 75, and 100 nm) and (b) a change in the NCs concentration for the same NC size, *L* = 100 nm. The AIPhP resonance moves to lower frequencies as the NC size decreases, while the amplitude of the response is only affected by the different concentrations. Each resonant peak is marked with a corresponding dashed vertical line. *L* is the NC dimensions.

Finally, we compute the mode volume and quality factor of the AIPhP resonators using the quasi-normal mode theory (see Methods) and compare the obtained results to those surface-plasmon AIPs in the visible spectrum and graphene-plasmons AIPs in the MIR spectrum. Figure 5a presents the calculated normalized mode volume, *V*_polariton_/λ_0_^3^, where λ_0_ is the free-space wavelength, for each resonator with varying spacer thicknesses *d*.^20,27^ It can be seen that our AIPhP resonator achieves a normalized mode volume approximately 2 orders of magnitude smaller than that of the surface-plasmon AIPs, reaching a value of 10^–8^ for wavelengths that are over an order of magnitude larger. We note that while the graphene-plasmon AIPs exhibit smaller normalized mode volumes compared to the AIPhPs, their resonances reside at shorter wavelengths.

**Figure 5:**
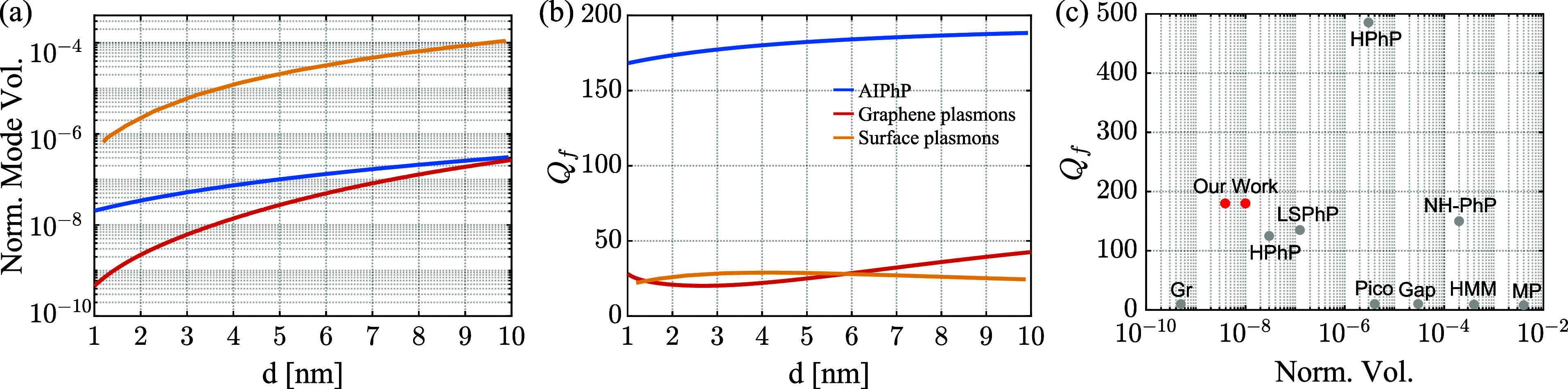
(a) Normalized mode volume and (b) quality factor calculations as a function of the spacer thickness *d* for AIPhPs (blue curve), graphene-plasmon AIPs (red curve), and surface-plasmon AIPs (orange curve) resonators. (c) Comparison to other types of resonators in terms of *Q* factors vs normalized cavity volume following ref 63: picocavities (Pico),^64^ nanogap plasmon polaritons (Gap),^65^ GPP (Gr),^27^ metallic particles (MP),^66^ hyperbolic metamaterials (HMM),^67^ HPhP,^63^ localized SPhP (LSPhP),^43^ and nonhyperbolic PhP (NH-PhP).^68^ The two AIPhP points correspond to the calculated and physical mode volumes.

Respectively, in Figure 5b, we present the calculated *Q* factor of the resonators as a function of the spacer thickness. Notably, our AIPhP resonators achieve *Q* factors that are over an order-of-magnitude larger than the equivalent resonators, demonstrating its capacity to store a significantly larger amount of energy due to the reduced losses provided by SPhPs.^69^ For completeness, we also compare our results with other types of resonators; however, because mode volume calculations of these are sparsely found in the literature, we follow the approach and terminology of Herzig Sheinfux et al.,^63^ taking the approximated mode volume as the physical volume of the resonator. The comparison is presented in Figure 5c, showing that our AIPhP resonators can achieve extremely small mode volumes with relatively large *Q* factors.

In this study, we have measured and observed the far-field excitation of AIPhP resonators in SiC. We have demonstrated that each resonator exhibits remarkably small normalized mode volumes with low losses and with a spectrally controllable and tunable response. This was achieved by depositing silver NCs on top of a SiC surface, with each NC functioning as an AIPhPs resonator. This versatile platform may open the path for controlling and manipulating strong light–matter interactions at the nanoscale in the long-wavelength range.

## Methods

### Sample Fabrication

The 4H semi-insulating 500-μm-thick SiC wafers were obtained from MSE Supplies LLC and subsequently coated with single layers of poly­(allamine hydrochloride) (Sigma-Aldrich), followed by the deposition of silver NCs (NanoComposix), as described in ref 70.

### Optical Measurements

The far-field reflectivity spectra of the samples were measured using a commercial FTIR (Bruker, Vertex V70) coupled to a microscope (Bruker, Hyperion) equipped with a 15× objective with a spectral resolution of 1 cm^–1^. We conducted 400 scans for each FTIR measurement and smoothed each measurement using a Savitzky–Golay filter. The reflectance spectra reported were all in reference to a gold mirror.

### Simulations

Simulations of a single SiC/NC resonator were conducted to investigate nonperiodic isolated objects utilizing the rigorous coupled-wave analysis method. This method, originally developed for periodic structures, was adapted for our specific application.^61^ The computation of poles in omega and the normalization of quasi-normal modes (QNMs) are based on perfectly matched layers as nonlinear coordinate transforms.^71^ Definitions of QNMs and modal volume were derived from relevant literature.^72^

## Supplementary Material



## Notes

Additional SEM images and simulation description (PDF)
