# Supplementary material for: Nanometer-Scale Cavities for Mid-Infrared Radiation via Image Phonon Polariton Resonators
Source: Nano Lett. 2025 May 19;25(22):8999–9005. doi: 10.1021/acs.nanolett.5c01352 (PMC12142678; doi:10.1021/acs.nanolett.5c01352)
Supplement: Supplementary file 1 [file nl5c01352_si_001.pdf]

Supplementary Information
Nanometer-scale Cavities for Mid-infrared Radiation via Image Phonon Polariton Resonators

Michael Klein,[†] Yonatan Gershuni,[†] Alisa Perutski,[†] Jean-Paul Hugonin,[‡] and Itai Epstein[*,†,¶,§]

†*School of Electrical Engineering, Faculty of Engineering, Tel Aviv University, Tel Aviv 6997801, Israel*

‡*Universite Paris-Saclay, Institut d'Optique Graduate School, CNRS, Laboratoire Charles Fabry, 91127 Palaiseau, France*

¶*Center for Light-Matter Interaction, Tel Aviv University, Tel Aviv 6997801, Israel*

§*QuanTAU, Quantum Science and Technology Center, Tel Aviv University, Tel Aviv 6997801, Israel*

E-mail: itaieps@tauex.tau.ac.il

Fill-factor calculations

Higher magnification images were obtained using a scanning electron microscope (SEM). The analysis relies on high-contrast images to differentiate between the nanocubes (NC) and the substrate. The fill factor is calculated as the area covered by cubes divided by the area that is not. $Fillfactor = NC_{area}./Substrate_{area} * 100$

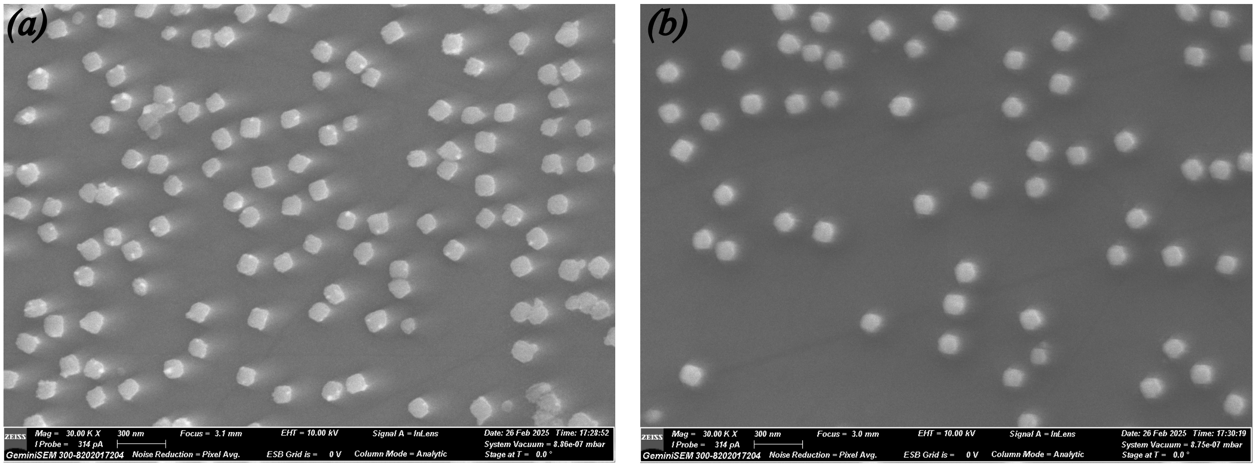

Figure 1: SEM images of two different concentrations, (a) high concentration, fill factor 13%, (b) low concentration, fill factor 5.8%

Simulations

The numeric calculations of fig.1 in the main test were performed based on the protocol.[1] The reflectivity calculations of Fig.2a in the main text are based on the transfer matrix method.

References

(1) Gershuni, Y.; Epstein, I. In-plane exciton polaritons versus plasmon polaritons: Nonlocal corrections, confinement, and loss. *Physical Review B* **2024**, *109*, L121408.
